# Trend of clinical drug trials in type 2 diabetes mellitus over last decade

**DOI:** 10.4103/2229-3485.80369

**Published:** 2011

**Authors:** Rakesh Parikh, Kirti Pandia, Mahesh Goyal, Meenakshi Sharma, M. S. Dolima

**Affiliations:** *D Clinarch, Unnati Tower, Vidhyadhar Nagar, Jaipur, India*

**Keywords:** Clinical drug trials, representation in clinical drug trials, trend of clinical drug trials, type 2 DM

## Abstract

**Background:**

Type 2 diabetes mellitus (Type 2 DM) has been recognized as the recent pandemic; India and China competing each other for the title—“Diabetes Capital of the World.” A number of new drugs have been recently available and has lead to a boom in the clinical drug trial industry. We intend to evaluate the trend of clinical drug trials in Type 2 DM over last one decade.

**Materials and Methods:**

Clinical drug trial registry of USA was used for getting the data regarding number of drug trials conducted in each country over last decade. India, China, and USA being the countries with highest prevalence of diabetes were included in the analysis. The percentage share of each country in clinical drug trials in Type 2 DM was compared with their percentage share in prevalence of Type 2 DM.

**Discussion:**

A significant growth in the drug trials in Type 2 DM was observed during 2005 to 2008, after which there has been a plateau. It was also recognized that India and China which contribute to around 30% of diabetic population of the world contributed in only 9.73% and 5.15% of drug trials in Type 2 DM during 2010, respectively. USA comprising of 15.15% of diabetic population of world was seen to have contributed in 38.36% of clinical drug trials in Type 2 DM. This raises a question of skewing in the data generated from various drug trials conducted in Type 2 DM.

## INTRODUCTION

Diabetes is the recent pandemic with the current estimated worldwide prevalence being 284.81 million.[1] India and China are both claiming to be the diabetes capital of the world. Last 5 years have witnessed a number of new therapeutic targets being explored and numerous new drugs have been introduced for the management of type 2 diabetes mellitus (Type 2 DM). In addition to India and China contributing for around 30% of diabetic population, United States of America (USA) has a high prevalence of diabetes. By virtue of its long-term complications that significantly add to the morbidity and mortality, diabetes has been recognized as one of the important threat to the health of society. The growing potential for pharmaceutical market has driven number of pharmaceutical companies toward clinical research in Type 2 DM. It is worthwhile looking at the trend of clinical drug trials in the field of Type 2 DM and the contribution of USA, India, and China in these trials. The new drugs being meant to be used in the whole globe, it would be appropriate to expect the fair representation of the so called diabetes capitals of world in the clinical drug trials meant for Type 2 DM. The trend of drug trials globally and in India, China, and USA was evaluated and their contribution in drug trials and that in prevalence of Type 2 DM were compared.

## MATERIALS AND METHODS

The total number of trials in Type 2 DM, the number of trials conducted each year during the last decade, and the number of trials conducted in above mentioned countries were obtained from the Clinical Drug Trials Registry of USA[2] using advanced search feature. Line chart of total number of trials in Type 2 DM, trials conducted in USA, China, and India during each year between 1999 and 2010 was plotted. The estimated prevalence of Type 2 DM worldwide and in the above mentioned countries was obtained from Diabetes Atlas of International Diabetes Federation.[1] Pie chart was plotted so as to have a visual impression of contribution of these countries in the world diabetes population.

## DISCUSSION

**Figure 1:**
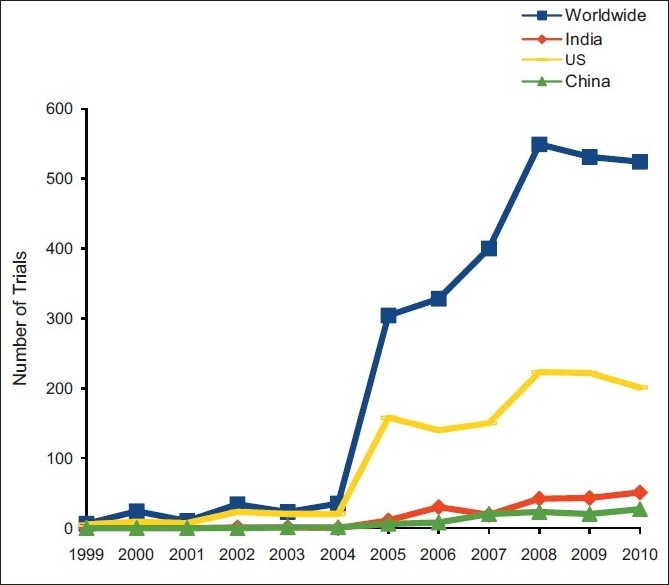
Trend of clinical drug trials in type 2 DM during 1999–2010

Year 1999 to 2004 witnessed a slow growth in the clinical trial industry, the number of trials in diabetic population being 6 in 1999 and 35 in 2004. There was significant spike during the year 2005, the total number trials shooting up to 304. A steady growth was maintained till the year 2008, after which there has been a plateau in the growth reflected throughout the countries [Figure 1].

**Figure 2:**
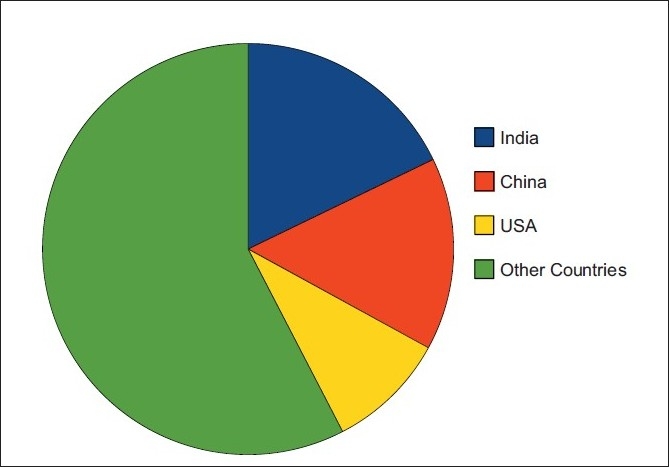
Contribution of India, China, and USA in estimated global prevalence of Type 2 DM during 2010 (Global Prevalence being 284.81 million)

The analysis of estimated global prevalence of diabetes during 2010 revealed that India and China contributed to around one-third of diabetic population of the world. Of 284.81 million diabetic subjects worldwide, India and China contribute 50.77 and 43.16 million patients, respectively, the prevalence in USA being 26.81 million. Rest of the world accounted for 164.07 million diabetic subjects[Figure 2].

**Figure 3:**
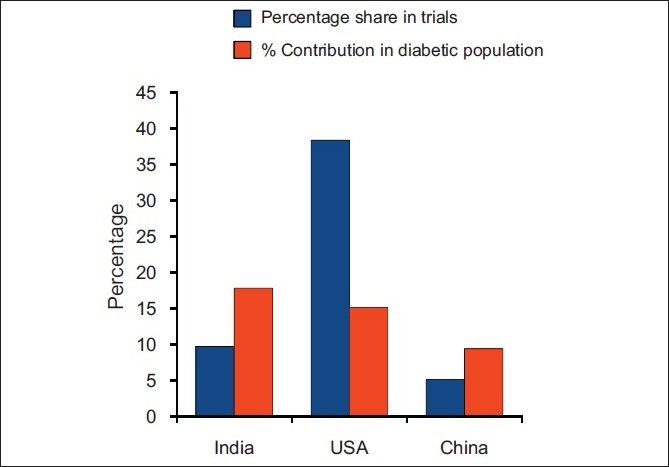
Percentage contribution in prevalence of diabetes and percentage share in clinical drug trials in Type 2 DM

While analyzing the percentage contribution of each country in clinical trial in Type 2 DM in respect to their percentage contribution in the prevalence of Type 2 DM, it was observed that though India and China had comparable representation, USA grabbed the major share in clinical drug trials. India and China having 17.83% and 15.15% of world diabetic population were involved in 9.73% and 5.15% of clinical drug trials in diabetic subjects, respectively. However, USA with 9.41% of diabetic population had 38.36% share in drug trials in diabetic subjects[Figure 3].

This observation raises a concern that the results of most of the clinical drug trials might be skewed due to proportionately higher number of representation of USA population, though India and China are the biggest market for these drugs. Whether the skewed data obtained largely from USA population can be applied to India and China is a question that needs further evaluation.
